# Bioinformatics role of the WGCNA analysis and co-expression network identifies of prognostic marker in lung cancer

**DOI:** 10.1016/j.sjbs.2022.02.016

**Published:** 2022-02-23

**Authors:** Liang Chengcheng, Sayed Haidar Abbas Raza, Yu Shengchen, Zuhair M. Mohammedsaleh, Abdullah F. Shater, Fayez M. Saleh, Muna O. Alamoudi, Bandar H. Aloufi, Ahmed Mohajja Alshammari, Nicola M. Schreurs, Linsen Zan

**Affiliations:** a College of Animal Science and Technology, Northwest A&F University, Yangling, Shaanxi 712100, PR China; b National Beef Cattle Improvement Center, Northwest A&F University, Yangling, Shaanxi 712100, PR China; c Department of Medical Laboratory Technology, Faculty of Applied Medical Sciences, University of Tabuk, Tabuk 71491, Saudi Arabia; d Department of Medical Microbiology, Faculty of Medicine, University of Tabuk, Tabuk 71491, Saudi Arabia; e Biology Department, Faculty of Science, Hail University, Hail 81411, Saudi Arabia; f Animal Science, School of Agriculture and Environment, Massey University, Palmerston North, New Zealand

**Keywords:** Bioinformatics, Lung Cancer, Gene Expression Omnibus, Weighted Correlation Network Analysis (WGCNA), Gene Expression Profiling Interactive Analysis (GEPIA)

## Abstract

Lung cancer is the most talked about cancer in the world. It is also one of the cancers that currently has a high mortality rate. The aim of our research is to find more effective therapeutic targets and prognostic markers for human lung cancer. First, we download gene expression data from the GEO database. We performed weighted co-expression network analysis on the selected genes, we then constructed scale-free networks and topological overlap matrices, and performed correlation modular analysis with the cancer group. We screened the 200 genes with the highest correlation in the cyan module for functional enrichment analysis and protein interaction network construction, found that most of them focused on cell division, tumor necrosis factor-mediated signaling pathways, cellular redox homeostasis, reactive oxygen species biosynthesis, and other processes, and were related to the cell cycle, apoptosis, HIF-1 signaling pathway, p53 signaling pathway, NF-κB signaling pathway, and several cancer disease pathways are involved. Finally, we used the GEPIA website data to perform survival analysis on some of the genes with GS > 0.6 in the cyan module. CBX3, AHCY, MRPL12, TPGB, TUBG1, KIF11, LRRC59, MRPL17, TMEM106B, ZWINT, TRIP13, and HMMR was identified as an important prognostic factor for lung cancer patients. In summary, we identified 12 mRNAs associated with lung cancer prognosis. Our study contributes to a deeper understanding of the molecular mechanisms of lung cancer and provides new insights into drug use and prognosis.

## 1 Introduction

Cancer is defined as an abnormal growth of cells. Cancer originates in any organ or body structure and consists of tiny cells that have lost the ability to stop growing. The incidence of cancer has been rising steadily over the past decade, probably due to our regular exposure to various carcinogens, or cancer-causing agents, and changes in our lifestyles. Cancer is one of the most dreaded diseases of the 20th century and is spreading further in the 21th century as the incidence continues and increases. This is a very worrying situation, with one in four people at risk of developing cancer (Roy and Saikia, 2016, Torre et al., 2016). Cancer profiles are inseparable from their internal metabolism, whether the cancer profile leads to altered internal metabolism or the reverse. Alterations in signal transduction pathways may explain the metabolic reprogramming of cells (Kroemer and Pouyssegur, 2008). Cancers with lethal potential, or their precursors, should be detected early for treatment that reduces mortality and morbidity (Srivastava et al., 2019).

With increasing urbanization and environmental pollution, the causative factors of lung cancer have become increasingly complex. Research directions have focused on the epidemiological characteristics of lung cancer and its associated risk factors (Mao et al., 2016). Proteomics can detect relatively different protein abundances in normal and cancer patients, and analysis can identify predictive and prognostic markers of drug resistance in lung cancer. In turn, new molecular therapeutic targets can be identified (Cheung and Juan, 2017, Oberndorfer and Müllauer, 2018). Research on molecular markers of lung cancer is underway in Europe, which will potentially be an important screening tool (Veronesi, 2015).

WGCNA, or Weighted Correlation Network Analysis, is a measure of the co-expression relationship between genes using their expression correlation coefficients. Expression patterns between genes clustered into a module are similar. They can be involved in the same biological processes or signaling pathways (Liu et al., 2017, Kakati et al., 2019). In addition, these modules can also be associated with clinical features. When screening for differences, most studies focused on the differential genes and ignored the high correlation between genes. Currently, WGCNA has been applied in several research fields and is very effective for screening new therapeutic targets (Chen et al., 2017, Yuan et al., 2018, Yao et al., 2019).

In this study data were collected from the GEO dataset on the expression profile of lung cancer. Fifty-four of these samples (27 normal paraneoplastic normal tissues, 27 tumor cancerous tissues) were selected for data compilation. And the average FPKM < 0.5 expression was filtered. We then performed WGCNA analysis of the resulting genes and then constructed a protein interaction network on the highly correlated genes of lung cancer samples and survival analysis by combining the clinical data from GEPIA database, and screened a total of 12 genes related to survival rate. The screened genes could be used as prognostic markers of lung cancer for further study.

## 2 Methods

### 2.1 Data acquisition and pre‐processing

To screen for prognostic markers in lung cancer patients, this study used datasets from the GEO database, and GEO data contain expression profiling data and DNA methylation data.

### 2.2 Construction of Weighted Correlation Network Analysis

The value of mean FPKM was filtered for values greater than 0.5. We also perform cluster analysis on all samples and fit index analysis to convert the similarity matrix to adjacency matrix, and then we calculate the optimal power (soft threshold) value to ensure that the correlation between connectivity and power is above 0.9. The best power value was determined to be 14. Based on this, we constructed a scale-free network and a topology overlap matrix (TOM). Corresponding TOM dissimilarity (diss TOM) was also performed, which resulted in the generation of a gene (tree graph) hierarchical clustering tree based on function hclust by hierarchical clustering for module detection. To avoid generating too many modules, the relevant parameters were a minimum Module Size = 30 and deep Split = 2. MEDissThres = 0.25, the similarity is 0.75. When the similarity is > 0.75, the modules are merged to generate new merge module after that.

### 2.3 Relationship between grouping information and modules

The correlations between the constructed modules and the tumor and normal groups were analyzed, and a heat map plotted including 35 modules. Finally, we performed correlation analysis based on the gene significance of the module membership with the tumor group and plotted the scatter plot. The cyan module was found to have (Cor = 0.71, p-value = 1.4e-133). We further perform subsequent functional enrichment and analysis of this module.

### 2.4 Gene ontology and pathway enrichment analysis

The cyan module (Cor = 0.67, p-value = 3e-08) was screened and 200 hub genes were obtained by GS > 0.5 screening. Gene Ontology (GO) enrichment analysis of the hub genes was performed using the David version 6.8 (https://david.ncifcrf.gov/) database (Huang da et al., 2009). The kobas version 3.0 (http://kobas.cbi.pku.edu.cn/kobas3/?t=1) was selected for transformation of hub genes and Kyoto Encyclopedia of Genes and Genomes (KEGG) pathway enrichment analysis (Wu et al., 2006, Xie et al., 2011). The GO consists of three components: biological process (BP), molecular function (MF), and cellular component (CC). All displayed GO or KEGG terms or genes have a p < 0.05. Finally, we visualized some of the terms using the GO plot package (https://cran.r-project.org/web/packages/GOplot/) in the R language software (Walter et al., 2015).

### 2.5 Construction of PPI network

A protein interaction network map was constructed for the hub genes in the cyan module with GS > 0.5 (the higher the GS, the greater the correlation between the gene and the trait), which were displayed using the online website string (https://string-db.org/cgi/) (Szklarczyk et al., 2015, Szklarczyk et al., 2017). The screening condition was that with the highest confidence = 0.900. Further visualization was obtained using Cytoscape version 3.6.0 (https://cytoscape.org/) software (Shannon et al., 2003, Doncheva et al., 2019).

### 2.6 Survival analysis

Survival analysis was performed on some of the genes with GS > 0.6 in the cyan module, using the Gene Expression Profiling Interactive Analysis (GEPIA) website (Tang et al., 2017) (http://gepia2.cancer-pku.cn/# (index) of the Lung cancer disease clinical data for survival analysis of GS correlation>0.6 in the cyan module. The clinical sample consisted of 239 low expression groups and 239 high expression groups (S.Table 1).

## 3 Results

### 3.1 Pre‐processing of the data sets

We downloaded dataset GSE7670 and selected 54 of the samples (27 normal paracancerous normal tissues and 27 tumor cancerous tissues). The platform file used the GPL96 [HG-U133A] Affymetrix Human Genome U133A Array, and we performed id conversion of the matrix files according to the platform file. The matrix files were sorted and processed for missing values, and filtered for expression with an average FPKM < 0.5. We then performed WGCNA analysis of the resulting genes.

**Fig. 1:**
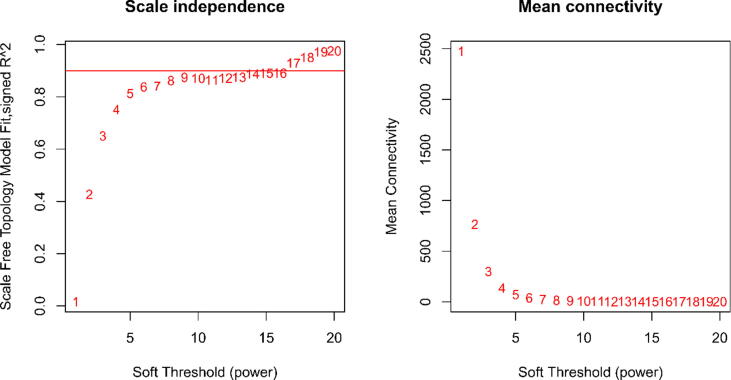
Scatter plot of fit index, average connectivity and power values. **Left:** Soft Threshold (power) is the weight, and the vertical axis indicates the correlation between connectivity and power. **Right:** Soft Threshold (power) is the weight, and the vertical axis is the average connectivity. The power when the correlation is required to reach 0.9 is used as the β value, β = 14 in this analysis.

**Fig. 2:**
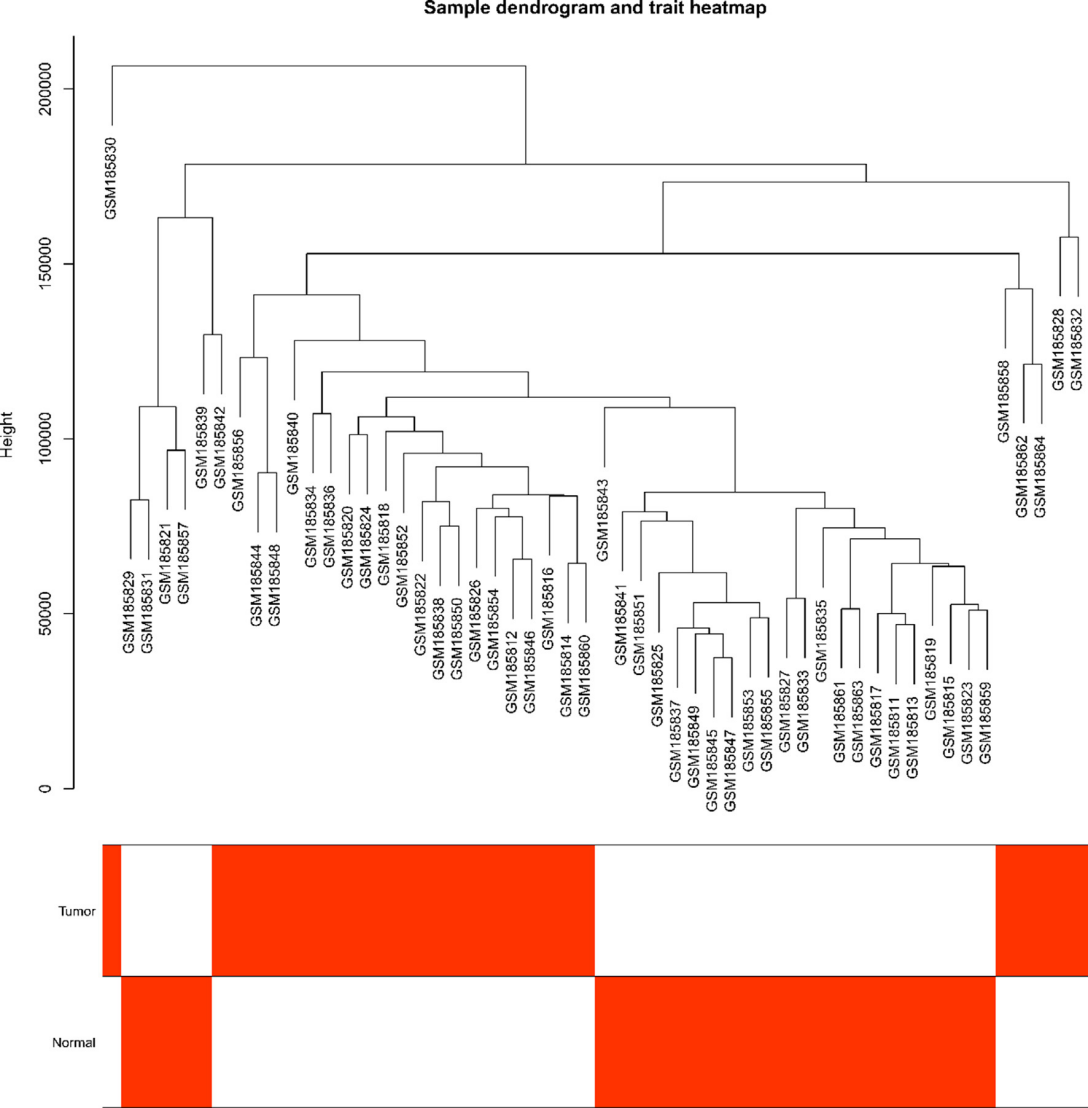
Sample clustering plot and sample trait heat map. The image is a clustering plot after clustering the samples, and below is a heat map of the samples corresponding to the traits Tumor and Normal, where red indicates the Tumor group. White indicates the Normal group.

**Fig. 3:**
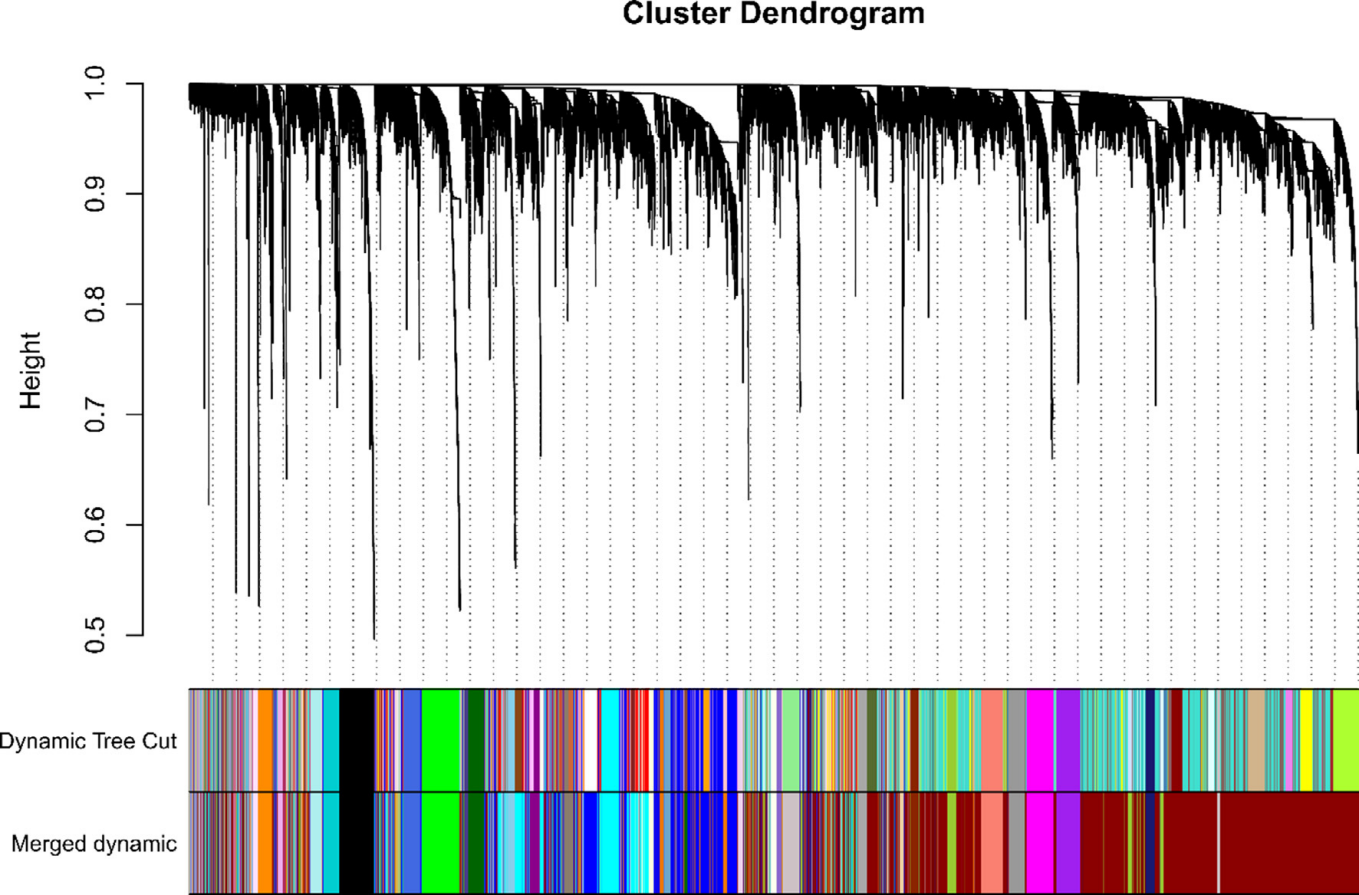
Module clustering tree diagram. Scale-free networks and topological overlap matrices (TOMs) were constructed. The corresponding TOM phase dissimilarity (diss TOM) was also performed, resulting in the generation of a gene (tree diagram) hierarchical clustering tree based on the function hclust by hierarchical clustering for module detection. In order to avoid generating too many modules, the relevant parameters min Module Size is 30 and deep Split = 2. MEDissThres is set to 0.25, the similarity is 0.75. When the similarity is > 0.75, the modules are merged together to generate new merge the module after that.

**Fig. 4:**
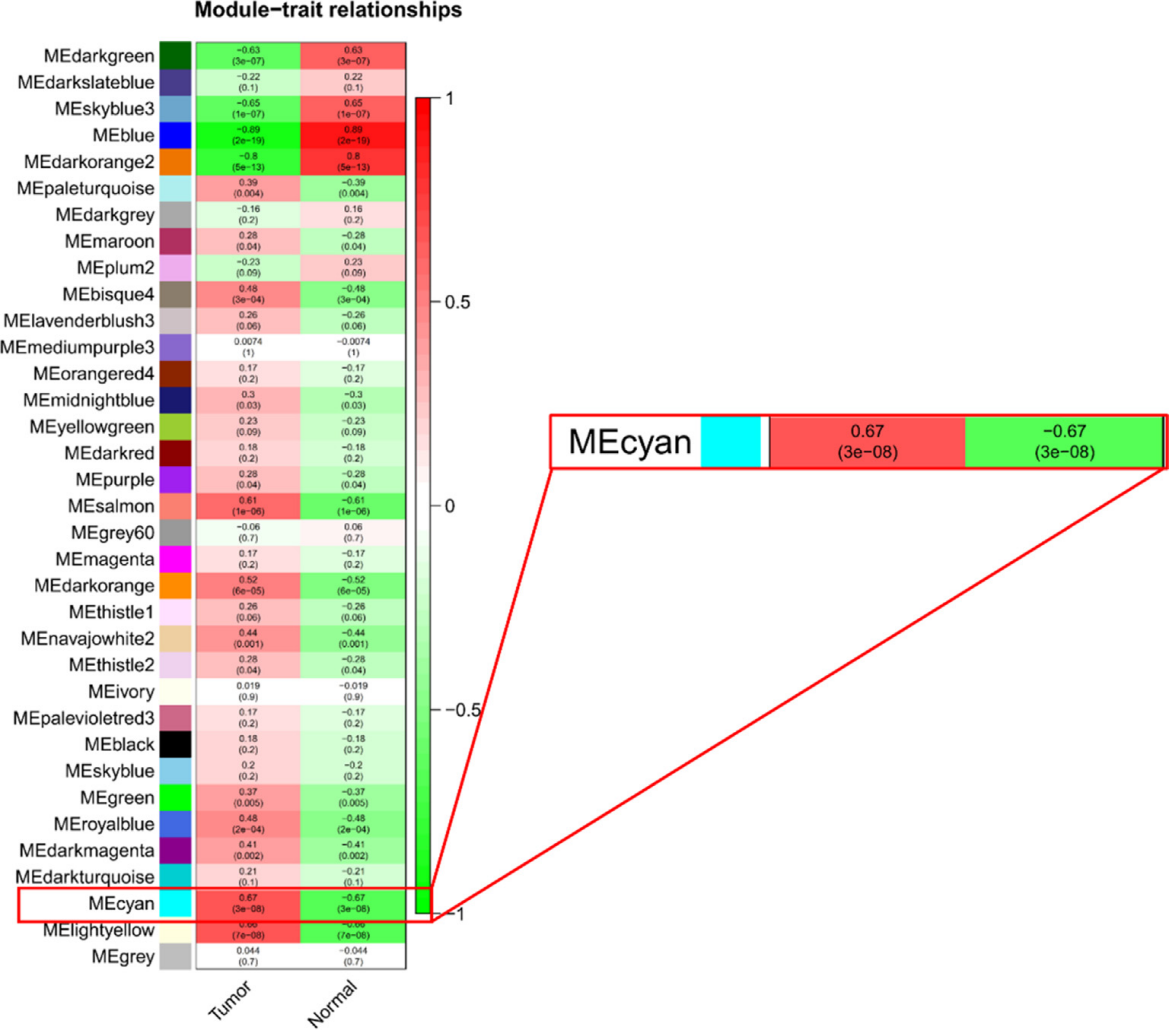
Module and trait-related heat map. The horizontal coordinates are traits, divided into tumor and normal groups. The vertical coordinates are the clustering obtained for each module. We chose the most correlated (Cor = 0.67, p-value = 3e-08) module cyan for the follow-up study.

**Fig. 5:**
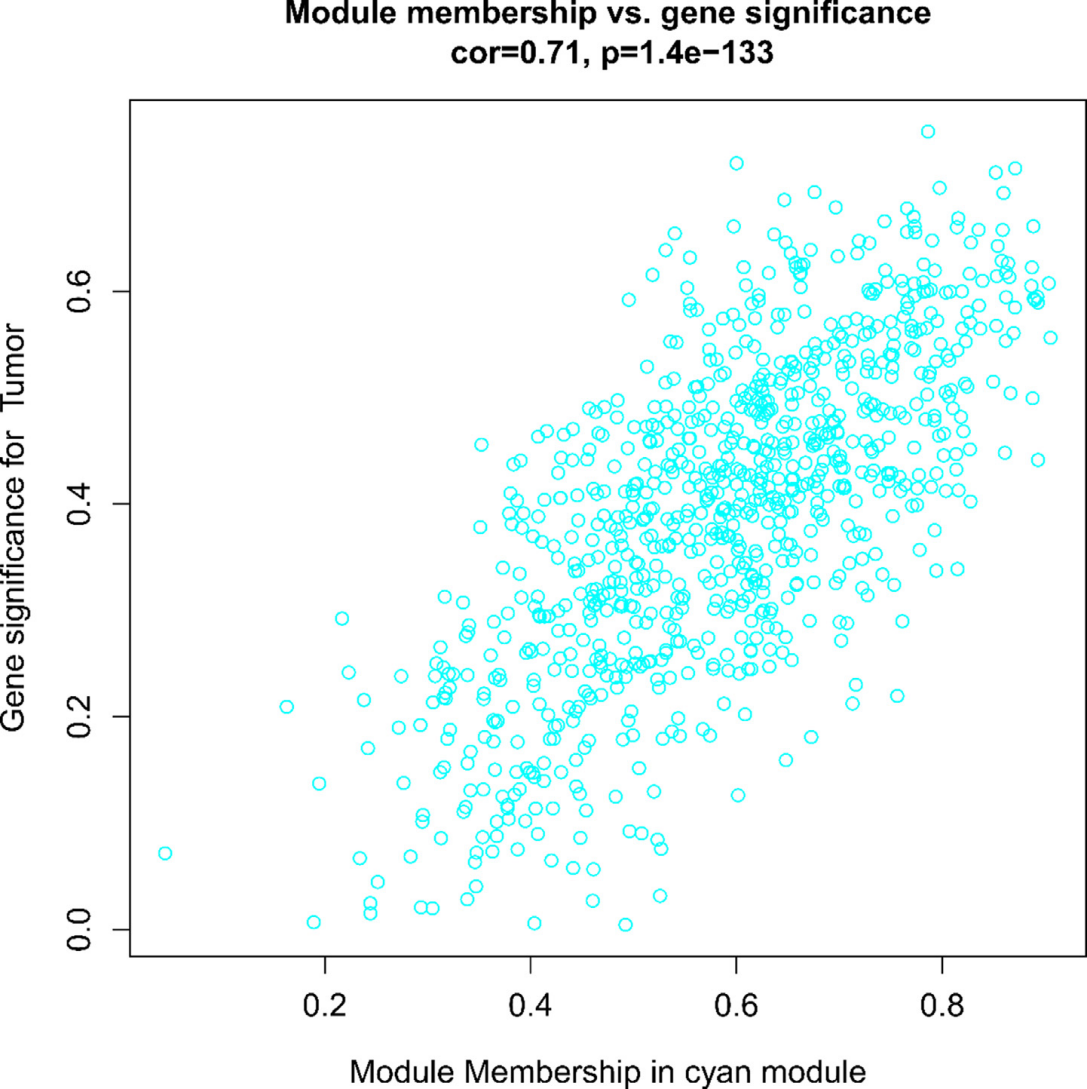
Scatter plot of correlation analysis of Cyan modules and tumor group. We can see that the correlation coefficient between Cyan module and tumor group reaches 0.71. To find the hub genes associated with the tumor trait, we first calculate the internal connectivity of the gene and the module, which measures the position of the gene within the module, while the module indicates to which module the gene belongs. The higher the correlation between the module MM and the current GS, the more positively the module is correlated with the trait tumor.

Fig. 1 shows the scale-free fit indices at various soft-threshold (power) values. There are plots showing the average connectivity at various soft-thresholds (power). The best power value was determined as β = 14. We then performed sample cluster analysis (Fig. 2). After grouping the adjacency matrices were transformed to generate TOM matrices, and hierarchical clustering was used to generate a function hclust hierarchical clustering-based tree for module detection. Dynamic tree clipping method was used for module identification. And modules with similarity greater than 0.75 are merged. The final module clustering tree diagram was obtained (Fig. 3). We perform correlation analysis between the various modules and trait groupings generated by clustering. A heat map of the module and trait data is plotted (Fig. 4). It is total of 35 modules were generated for all samples. The correlation coefficients and p-values between each module and each trait are listed in the heat map. Finally, we selected the Cyan module for subsequent analysis. Because this module had the largest correlation coefficient with the tumor group (Cor = 0.67, p-value = 3e-08). This study also looked at the correlation between MM (module membership) in the cyan module and GS (gene significance) in the trait tumor (Fig. 5). The correlation was found to be 0.71, p-value = 1.4e-133. It means that the genes in the cyan module had a high positive correlation with the tumor cancer group. We then performed functional studies on the genes in the cyan module.

### 3.2 Enrichment analysis of GO, KEGG and PPI network

**Fig. 6:**
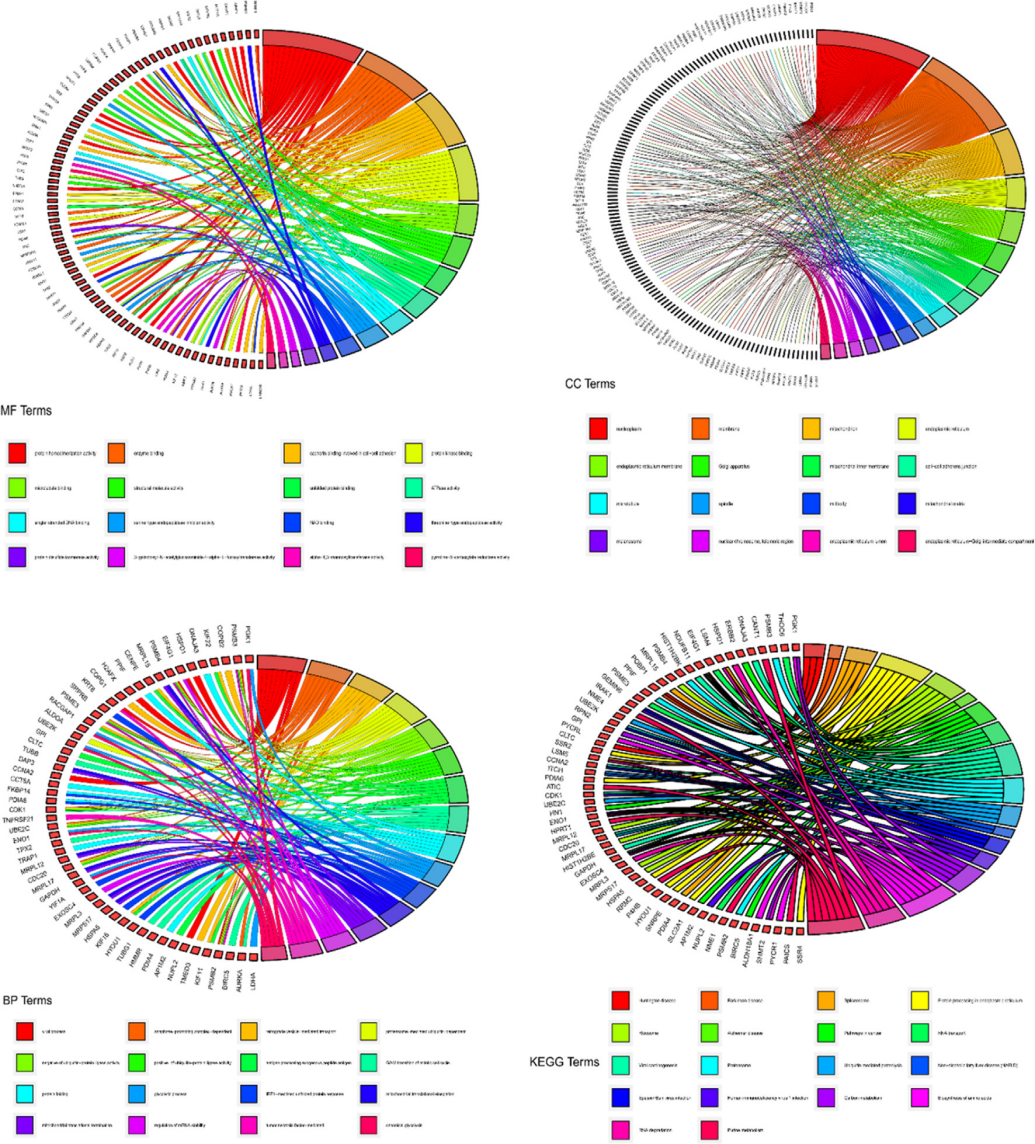
Chord diagram of GO and KEGG enrichment analysis of the Hub gene. GO enrichment analysis includes biological process (BP), molecular function (MF) and cellular component (CC) terms) and KEGG pathway enrichment analysis. Selected sections are presented.

**Fig. 7:**
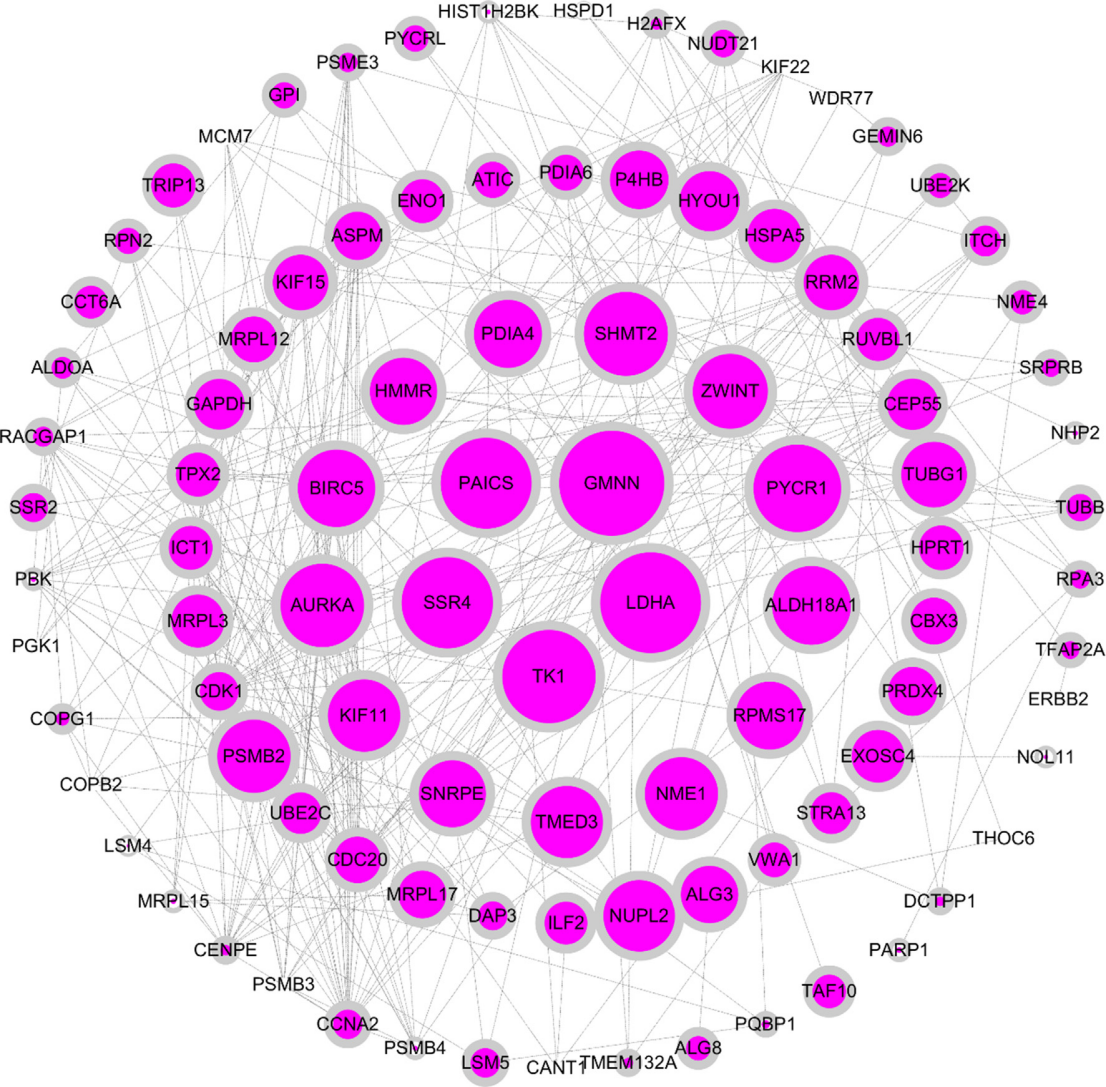
PPI protein interaction network diagram for the Hub gene. The protein interaction network map was constructed for the hub gene with GS > 0.5 in the cyan module, and the screening condition was highest confidence = 0.900. The size of nodes indicates the correlation between GS and tumor trait, and the larger the nodes, the stronger the correlation.

A total of 120 GO terms were found. Of these, 62 were biological processes (BP), 38 were cellular components (CC), and 20 were molecular functions (MF). Most of them were focused on cell division, tumor necrosis factor-mediated signaling pathway, negative regulation of cell redox homeostasis, negative regulation of reactive oxygen species biosynthesis, positive regulation of Wnt signaling pathway and other processes related to cancer cell division, maintenance of cell redox homeostasis and reactive oxygen species biosynthesis. KEGG pathway enrichment analysis enriched a total of 110 pathways, mainly including cell cycle, cellular apoptosis, HIF-1 signaling pathway, peroxisome, insulin resistance, PI3K-Akt signaling pathway, p53 signaling pathway, NF-κB signaling pathway, and multiple cancer disease pathways. We selected some of the results of GO and KEGG analyses and plotted them visually using the R package GO plot (Fig. 6). Finally, we performed protein interaction network analysis based on the genes with GS > 0.5 in the cyan module (Fig. 7). A total of 96 nodes and 311 edges were obtained. The size of the circles in the graph indicates the size of the correlation with GS tumor. The larger the circles, the stronger the correlation.

### 3.3 Survival analysis

**Fig. 8:**
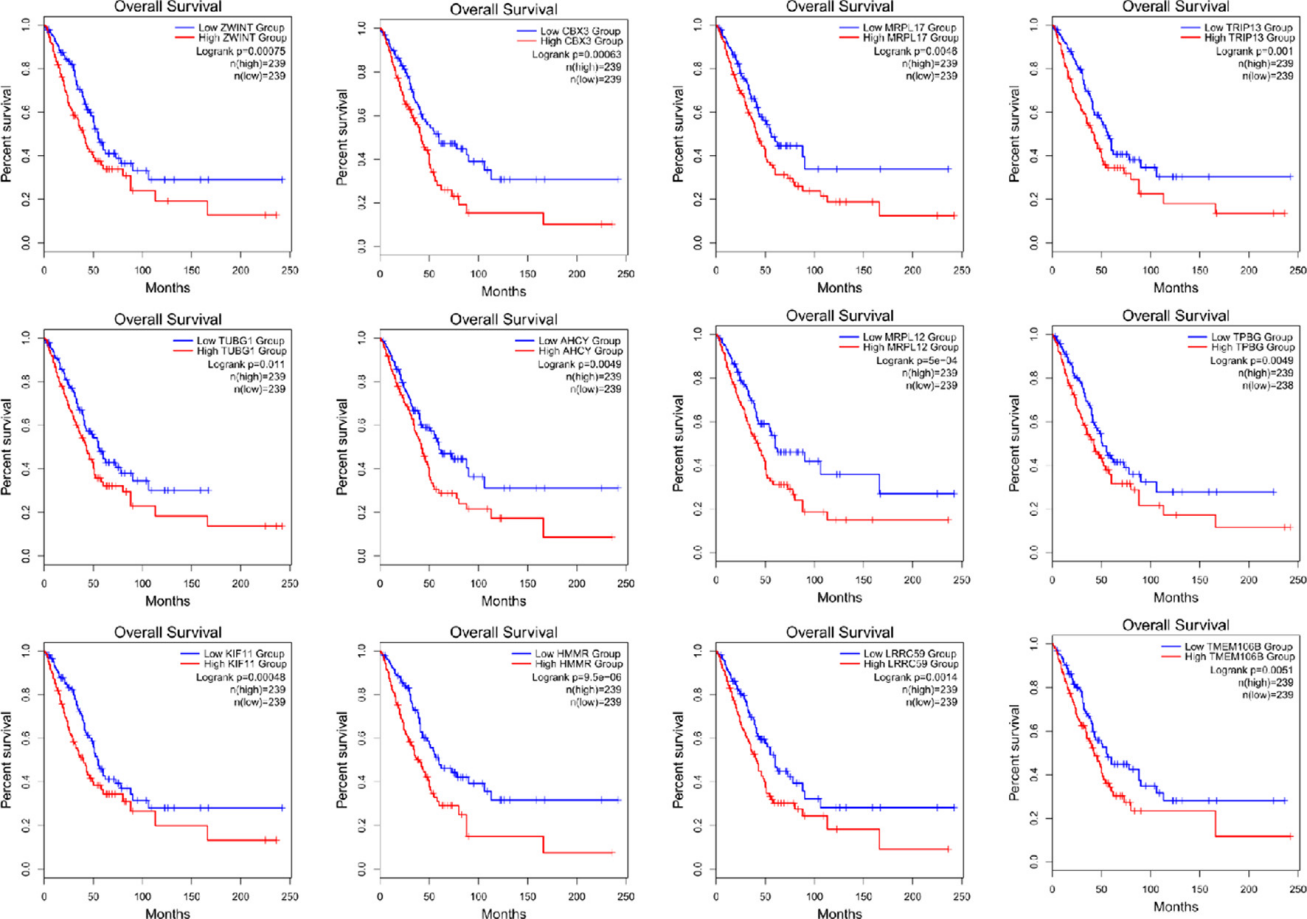
Survival analysis curves for selected Hub genes. GS correlation in the cyan module was greater than 0.6 for survival analysis, and the clinical sample consisted of 239 low expression groups and 239 high expression groups, method selection overall survival, using median's group cutoff, log rank p < 0.05.

A total of 12 lung cancer prognostic markers were screened: Chromobox 3 (CBX3), adenosylhomocysteinase (AHCY), Kinesin Family Member 11 (KIF11) by survival analysis based on the GEPIA public database, Mitochondrial Ribosomal Protein L12 (MRPL12), Leucine Rich Repeat Containing 59 (LRRC59), Hyaluronan Mediated Motility Receptor (HMMR), Mitochondrial Ribosomal Protein L17 (MRPL17), Transmembrane Protein 106B (TMEM106B), Trophoblast Glycoprotein (TPBG), Tubulin Gamma 1 (TUBG1), ZW10 Interacting Kinetochore Protein (ZWINT), Thyroid Hormone Receptor Interactor 13 (TRIP13). The survival analysis curve is shown in Fig. 8. When the expression of 12 genes is high, the survival rate of lung cancer patients is small; therefore, these genes may be associated with a poor prognosis.

## 4 Discussion

WGCNA analysis is more widely used in cancer research. Examples include studies related to colon cancer (Zhai et al., 2017), liver cancer(Yin et al., 2018) and osteosarcoma metastasis(Tian et al., 2018) This analysis allows for the classification of co-expressed genes by weighted gene co-expression. Listed as a module, each module and related traits are finally correlated, thus selecting module genes that are highly correlated with the trait for further analysis.

The incidence of lung cancer now leads the world and has gone from being a rare disease to a global public health problem. The causes of the disease have also become more complex than before. Most of these are due to smoking, environmental pollution problems, and industrialization. (Mao et al., 2016, Nasim et al., 2019). But with advances in the life sciences. New molecular targets for lung disease continue to be discovered, prompting the development of new therapies. For NSCLC, future use of targeted therapies or immunotherapy may be more effective (Hirsch et al., 2017).

Gene CBX3 mainly plays a role in neural differentiation and the growth of hepatocellular carcinoma cells, and has minimal studies for its association with lung cancer (Huang et al., 2017, Zhong et al., 2019). AHCY is associated with stem cell proliferation (Aranda et al., 2019), and studies on lung cancer are currently unreported. MRPL12 is mainly associated with mitochondrial energy supply (Ma et al., 2020)and the gene LRRC59 is reported to be associated with poor prognosis in lung cancer and is involved in the cell proliferation process (Li et al., 2020). The gene TPGBG is associated with Wnt signaling and can promote pancreatic ductal adenocarcinoma cell metastasis (He et al., 2015). The TUBG1 gene has been associated with the risk of breast cancer (Blanco et al., 2015). The gene we screened is not common in lung cancer studies. However, it has a high positive correlation with lung cancer traits. Therefore, it is of great research significance.

This is consistent with the characteristics of the sample and bodes well for the desirability of our research method. The results are reliable.

In this study, we used bioinformatics WGCNA analysis for modular analysis of lung cancer samples, and we identified 12 mRNAs that can be used as prognostic markers for lung cancer analysis. They included 5 unknown (CBX3, AHCY, MRPL12, TPBG, TUBG1) and 7 known (KIF11, LRRC59, MRPL17, TMEM106B, ZWINT, TRIP13, HMMR) markers.

## 5 Conclusion

Overall, we screened 12 mRNAs associated with prognosis in lung cancer that could serve as prognostic biomarkers for lung cancer research. The screening study performed contributes to further understanding of the molecular mechanisms of lung carcinogenesis and provides new insights into drug use and prognosis.

## Declaration of Competing Interest

The authors declare that they have no known competing financial interests or personal relationships that could have appeared to influence the work reported in this paper.
